# Engaging the stuff of words: language materiality and symbolic power

**DOI:** 10.1515/eduling-2024-0001

**Published:** 2025-10-21

**Authors:** Crispin Thurlow

**Affiliations:** Department of English and Center for the Study of Language and Society (CSLS), University of Bern, Bern, Switzerland; Wits Centre for Diversity Studies (WiCDS), University of the Witwatersrand, Johannesburg, South Africa

**Keywords:** language materiality, symbolic power, little “m” materialities, transmodalization, big “M” materialities, symbolic violence

## Abstract

A contribution from multimodality studies, this paper offers a practice- and teaching-oriented approach to the stuff of words. I start by introducing *language materiality*, a heuristic framework proposed by linguistic anthropologists, but grounding it in four allied precedents: *cultural studies*, *dispositif analysis*, *mediated discourse*, and *social semiotics*. Then, drawing on illustrative case studies from my own work, I demonstrate a two-part framework for working with students. In Part 1, the focus is on *little “m” materialities* and the transmodal interplay between words and things. In Part 2, the focus is on *big “M” materialities* and the more structural effects of language – specifically, symbolic violence. By using banal, everyday examples students can be helped to pinpoint the multimodality of words; how words function as material artefacts in their own right; and the way words materialize societal structures. In this way, students also learn to engage the stuff of words as more than just an analytical curiosity, but rather as an explicitly political-critical consideration.

## 1 Orienting to language materiality1Throughout, I italicize *language materiality* to signal its specificity as an analytic even though the inseparability of language and materiality function as an ontological reality too.

What creates the power of words and slogans, a power capable of maintaining or subverting the social order, is the belief in the legitimacy of words and of those who utter them. And words alone cannot create this belief. (Bourdieu 1991: 170)

In his famous treatise on language and symbolic power, Pierre Bourdieu (quoted above) makes some key observations which serve as the general rationale for the current paper. In short, words are never enough when it comes to understanding how social order is exercised and maintained; nor is language alone sufficient for explaining the power of words – words cannot and do not work alone. In many ways, this is the foundational principle of scholarship falling under the rubric of multimodality which, as Carey Jewitt (2009: 13) explains, “attends to the full range of communicational forms … and the relationships between them”. This words-are-not-enough principle has likewise helped direct applied linguists towards multimodality, as with Alastair Pennycook’s (2017) thinking about semiotic assemblages and Suresh Canagarajah’s (2018: 268) call for language instruction which properly recognizes the “material locus and spatiotemporal shaping” of communicative actions.2I am grateful to [one of the reviewers] for directing my attention to ways some applied linguists have been taking up so-called new materialism (e.g. Ennser-Kananen and Saarinen 2023; recall also Canagarajah 2018). My own take is that “new materialism” represents a well-intended correction but an over-correction nonetheless; as such, I follow scholars like Judith Butler (1993), Bruno Latour (2004) and Karen Barad (2003), who have long upheld the inseparability of words and things, and the inevitable interdependence of representational and non-representational meanings.


In concert with these theoretical principles and conceptual interventions, the current paper presents a practice- and teaching-oriented approach to the relationship between language and materiality – that is, to the stuff of words. Without providing “how-to” instructions or specific classroom tasks, I present a framework and indicative case-study examples. Working with seemingly banal, everyday texts can help students to pinpoint the multimodality of words; how words function as material artefacts in their own right; and the way words materialize societal structures of inequality. In this way, too, students can learn how to engage the stuff of words as more than just an analytical curiosity but rather as an explicitly political-critical consideration.

I begin the paper with two concrete examples for orienting to *language materiality*, a heuristic framework emerging from linguistic anthropology. I then briefly outline four other major precedents for studying the language–materiality relationship but which are overlooked by the language materiality framework; these are *cultural studies*, *dispositif analysis*, *mediated discourse*, and *social semiotics*. On the basis of this broader conceptual mapping, I then present a series of illustrative case studies drawn from my own work in order to consider (a) *little “m” materialities* and the transmodal interplay of words and things, and (b) *big “M” materialities* and the symbolic violence inherent in much everyday language. I conclude the paper by turning to waste as a quintessential example of language materiality at work in the world; I do this also by way of a concrete “trash talk” exercise designed to surface how storied matter matters.

**Figure 1:**
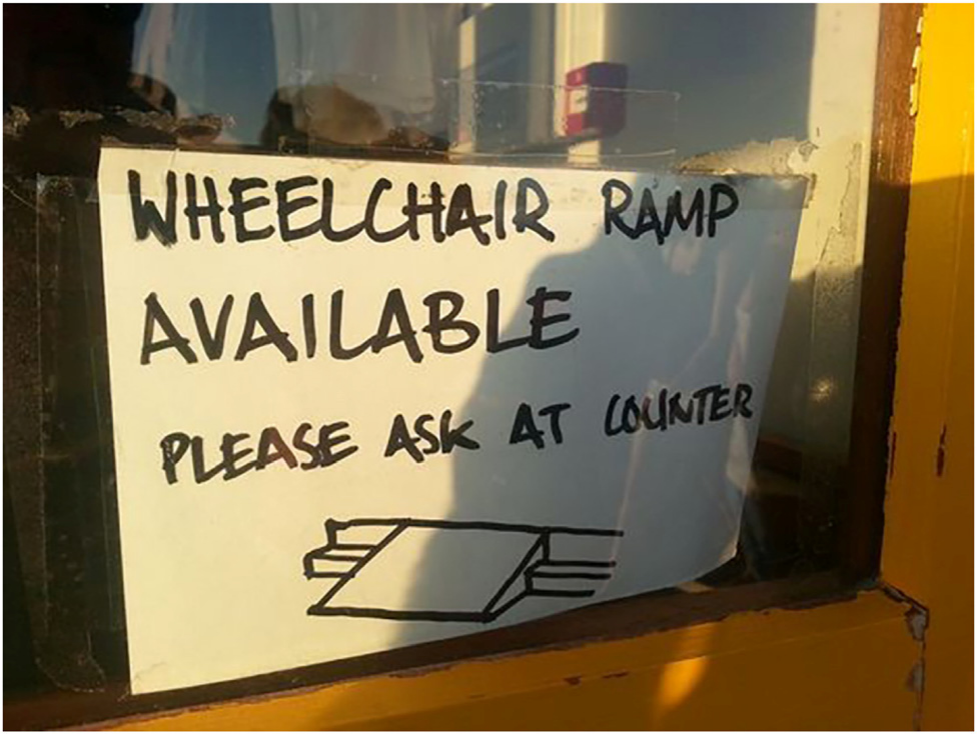
An *ad hoc* disability access notice (source unknown).

Figure 1 shows a good example of the banal, often innocently motivated ways in which *language materiality* functions in everyday life (see Thurlow 2022a). This is symbolic power in action through the combination of words, images, material artefacts, and spatial configurations. There is, of course, also power in the cultural, political, and societal structures conditioning this text. As such, we have here a little text (cf. Schmitz 2021) with big ramifications: small in size and tied to a specific purpose but manifesting large-scale social inequalities and power relations.

The image in Figure 1 shows a crudely handwritten notice carelessly sticky-taped to a window in which potential wheelchair users are told where to find a ramp for accessing the building. (I do not know the original source of the image, nor do I know the actual location depicted.) There is something inherently condescending about the sketch, as if a wheelchair user might not yet have grasped the meaning of the word “ramp”. The symbolic power is at its most condensed, however, in the deceptively convivial “available” and “please ask”. This is, I think, the worst kind of assimilationism (see Barnes and Mercer 2001). The author of the little text – probably abandoned by those with official responsibility for these matters – has patched together a notice which unwittingly animates some of the most deep-seated, fraught politics of disability access: an afterthought, casually arranged, and left to the disabled person to manage. All of which is done in the friendliest and, ironically, most accommodating way.

The point here is that the tangible materialities of this sign are important in and of themselves, but what is also significant is how they expose societal inequalities and injustices. The anthropologist Daniel Miller (2005: 19) sums things up nicely when he says, “[t]he study of material culture often becomes an effective way to understand power, not as some abstraction, but as the mode by which certain forms or people become realized, often at the expense of others”. In other words, there are two dialectically interdependent kinds of materiality at work in moments such as the one depicted in Figure 1: the materiality of things and embodied actions, and the materiality of structures and political economies.

**Figure 2:**
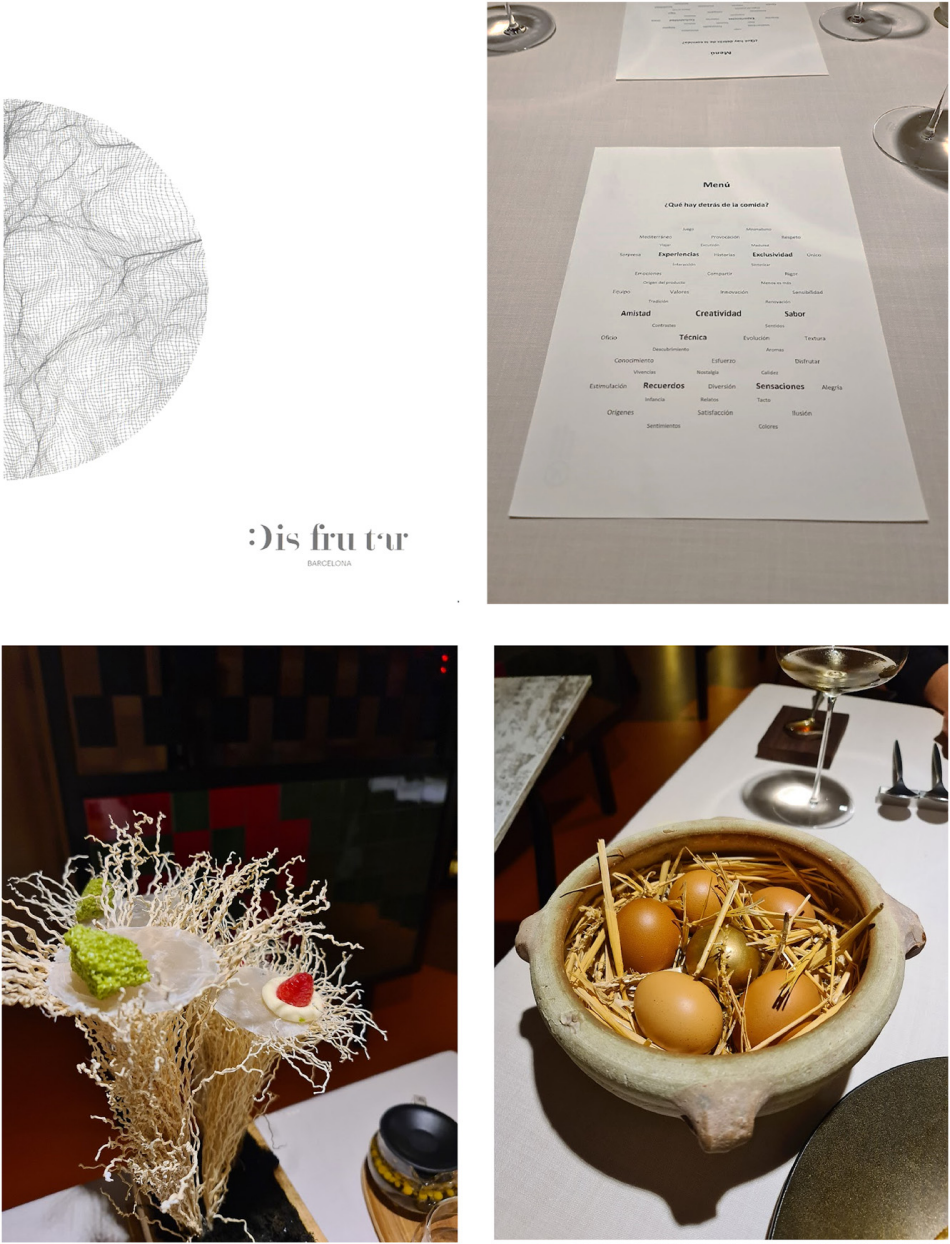
(a–d) Eating at *Disfrutar –* words, food, things (photos mine).

My second orienting example (Figure 2a–d) is a somewhat more spectacular case but, in principle at least, another typical instance of *language materiality* in contemporary life. In 2022, I was taken by a wonderful colleague to a very famous restaurant in Barcelona called *Disfrutar*. I never ordinarily eat in places like this, but *Disfrutar* has repeatedly been ranked as one of the top restaurants in the world. It turned out to be an extraordinary (and extraordinarily privileged) experience – both delicious and fascinating.

Shown in Figure 2a is the cover of the restaurant’s more conventional menu which we were given only at the end of the meal and only upon request. Instead, the start of the meal was signaled – and materialized – with the “menu” shown in Figure 2b. Under the heading *Qué hay detrás de la comida?* (‘what lies behind the food?’), we were offered this word cloud of “experiences”, “sensations”, “memories” and other abstract notions or ideas. As soon as this sheet was placed in front of me, I realized that whatever was about to happen in the way of our eating was going to be as much about the words as it was about the food. In fact, the whole meal – some 33 or 34 dishes – unfolded as a constant interplay between high materialities and a running, four-hour-long narrative by the waiters who outnumbered us three to one. It is not easy to recreate the performative spectacle of this meal, but the elaborate staging of the dishes is hinted at in Figure 2c and d. Each dish was presented with these kinds of theatrical props and platings, and always with a detailed, verbal account of the ingredients, their provenance, and their preparation.

The point I want to make is again a quite straightforward one: throughout the meal it was never quite clear whether it was the food itself – its spectacular presentation – or the constant verbal framing which was more meaningful. There was really no way of parsing this elaborate semiotic assemblage: the stuff and the words worked together to co-constitute (or co-signify) the meal. And this multimodal interplay is a cornerstone in the approach to language and materiality proposed by anthropologists Shalini Shankar and Jillian Cavanaugh (2012; also Cavanaugh and Shankar 2017) which they explain as follows:

Language and materiality have long been considered separate phenomena: the linguistic seen as inherently immaterial, the material as concrete and nondiscursive. Recent studies have featured an intriguing and productive convergence of the two that suggests the illustrative potential of considering language and materiality within the same analytic frame (Shankar and Cavanaugh 2012: 356)

**Figure 3:**
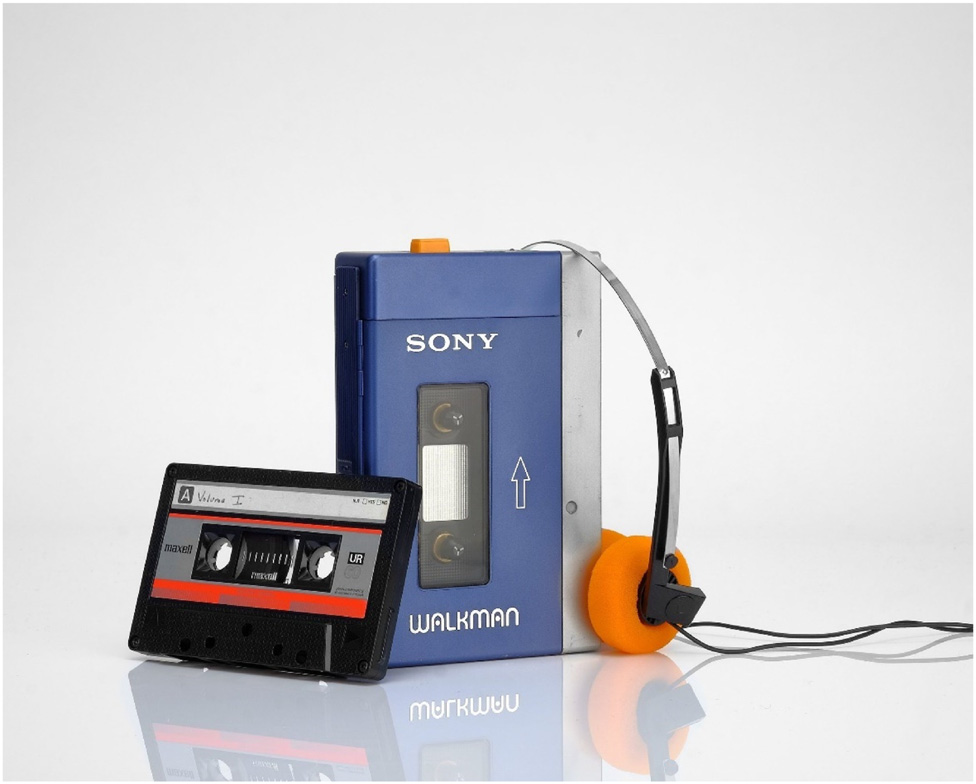
The *Sony Walkman* (source unknown).

**Figure 4:**
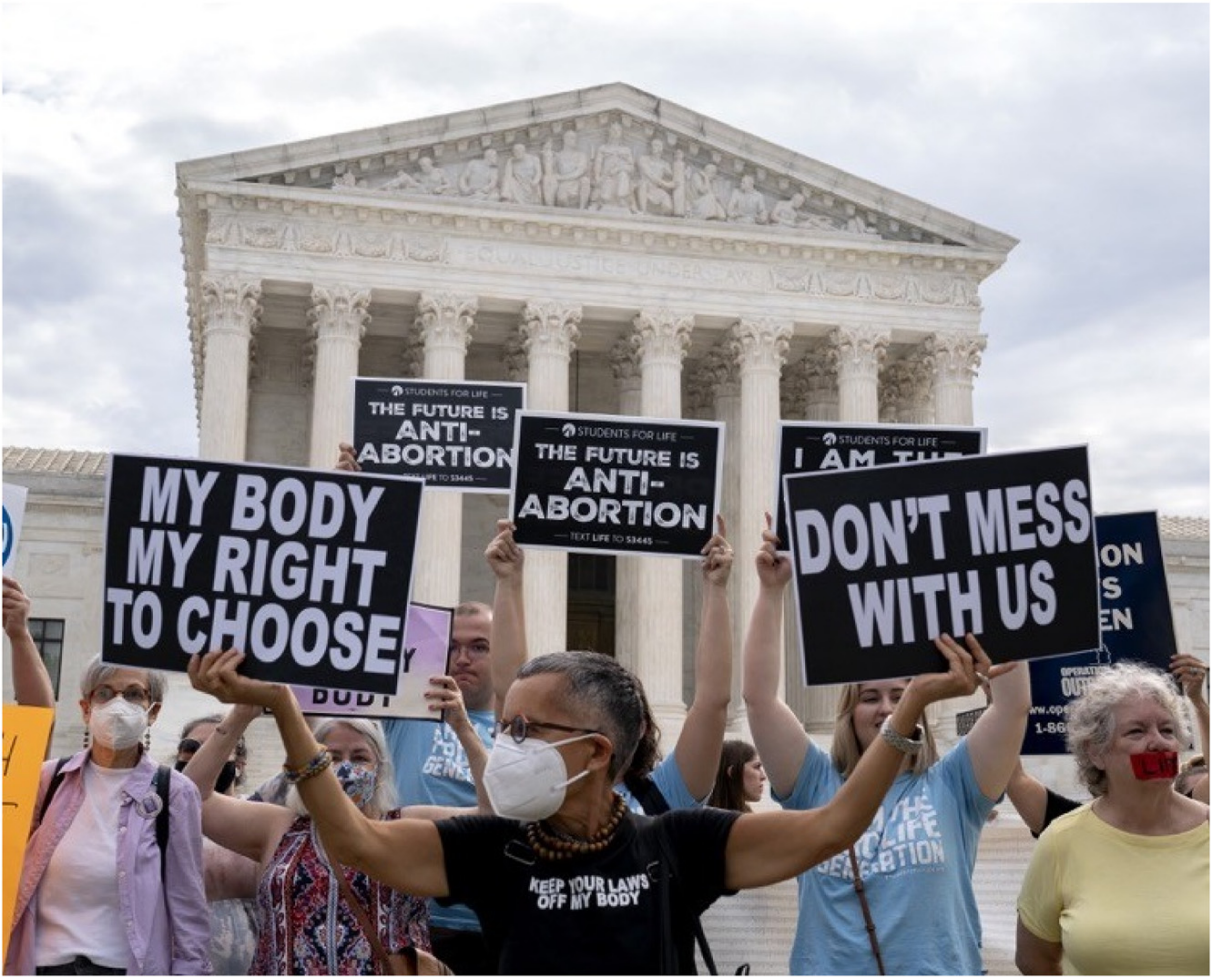
A Supreme Court (USA) protest (source unknown).

**Figure 5:**
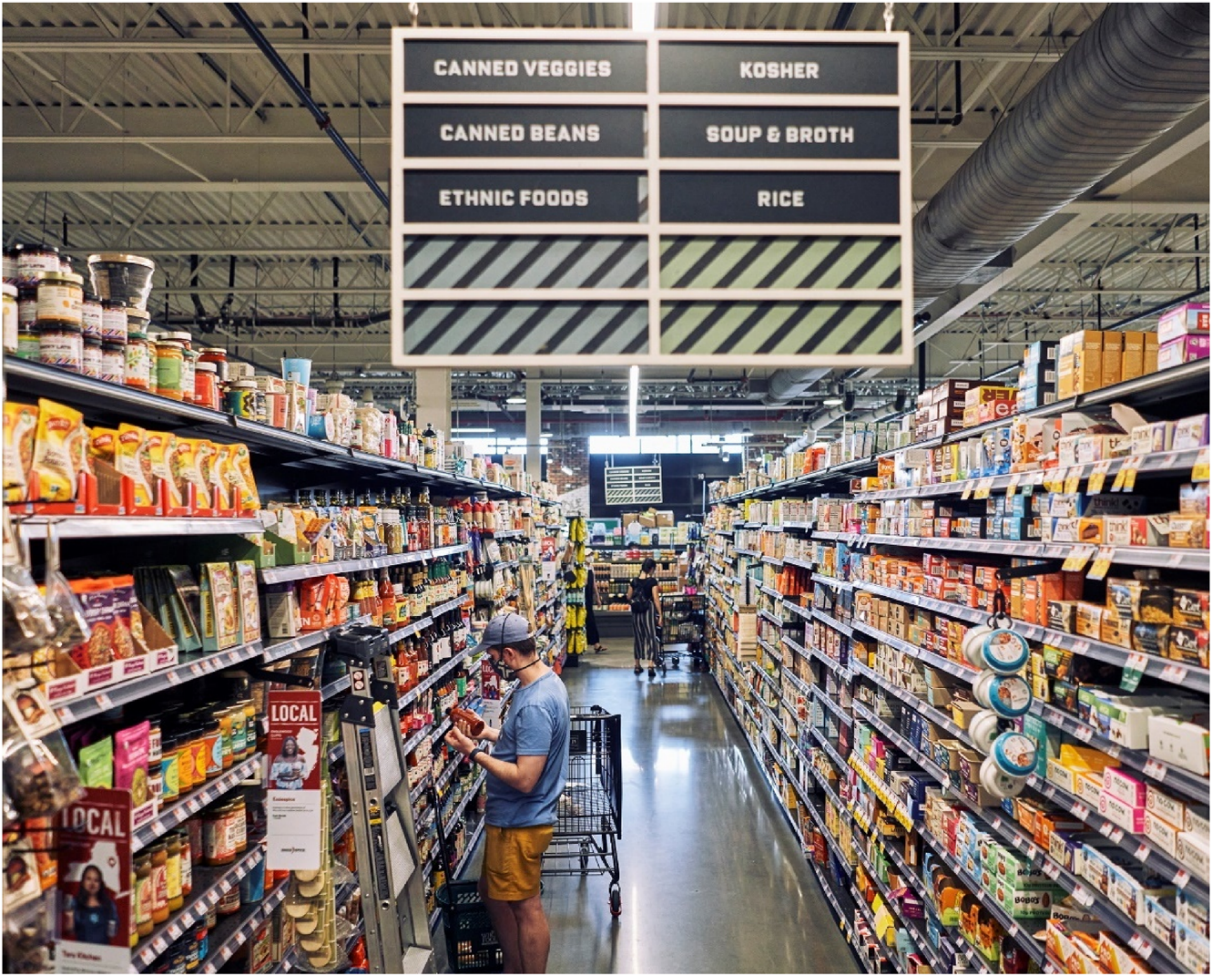
A supermarket aisle (source unknown).

**Figure 6:**
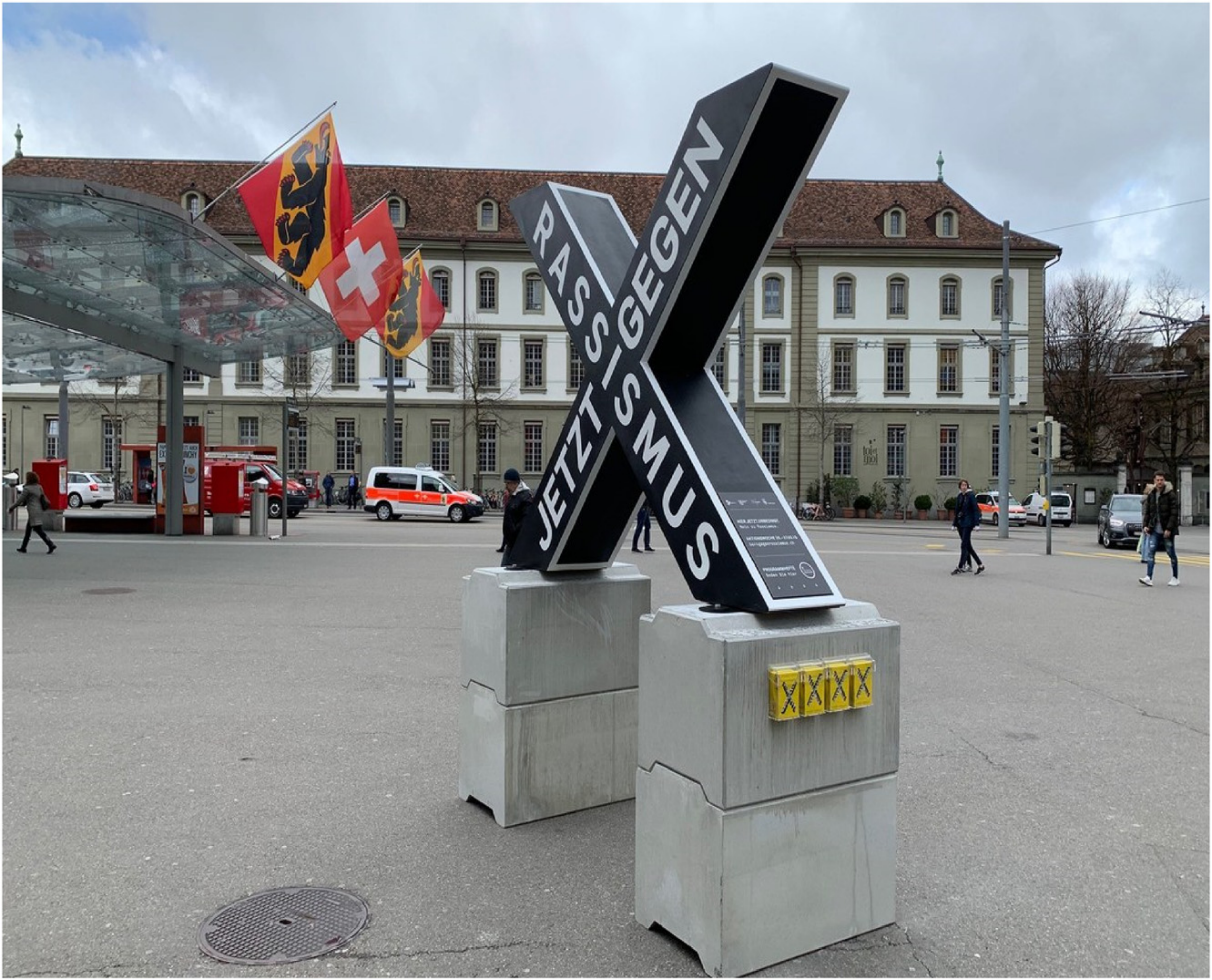
An anti-racism campaign in Bern (photo mine).

Such is the inevitable inseparability of linguistic and material practices that Shankar and Cavanaugh propose the label *language materiality* (without the “and”). The fundamental principle and critical intervention these scholars make is undeniably a sound one. As Karen Barad (2003: 828) had already argued, “discursive practices are not human-based activities but rather specific material (re)configurings of the world … [and] … matter is substance in its intra-active becoming – not a thing but a doing, a congealing of agency”. If anything, it is unfortunate that the *language materiality* framework overlooks these earlier theoretical insights as well as a number of other major methodological attempts to grapple with the relationship between language and materiality. In this regard, I want to call attention to four such precedents: *cultural studies*, *dispositif analysis*, *mediated discourse*, and *social semiotics*. By way of the practical examples illustrated in Figures 3 to 6, I offer just a brief snapshot of each of these allied approaches. To be clear, my selection here is neither comprehensive nor exhaustive; rather, I have selected these four approaches because they are closely aligned with sociocultural linguistics, which has always sought to to link small-scale practices with larger-scale social processes. The four allied approaches below also offer methodological – hence, practice-oriented – approaches to the analysis of textual, linguistic and communicative practices.

## 2 Language and materiality precedents

### 2.1 Cultural studies

Especially in the way that it emerged in Britain, cultural studies has always been an approach which fundamentally recognizes the need to situate the analysis of representational (or textual) practices in their material contexts. In this way, cultural studies scholars are also well-known for having expanded the scope of representation beyond written or spoken language to include a vast range of other types of popular cultural practices and artefacts. One of the most famous examples of this approach is Paul Du Gay et al.’s (1997) close analysis – or “reading” – of the now- quaint technology that was the Sony Walkman (Figure 3). In order to understand the social meaning of the Walkman – or any other text, practice, or artefact – five processes or contextual sites must be taken into consideration; together, these form what Du Gay et al. (1997: 3) refer to as the “circuit of culture” which they explain as follows:

… taken together [these five processes] complete a sort of circuit – what we term the circuit of culture – through which any analysis of a cultural text or artefact must pass if it is to be adequately studied. … one should at least explore how [the text or artefact] is represented, what social identities are associated with it, how it is produced and consumed, and what mechanisms regulate its distribution and use.

In this way, then, cultural studies starts from an understanding that material structures and other structuring practices – specifically production, consumption, and regulation – are central to the analysis of all representational practices, including language.

### 2.2 Dispositif analysis

Another sociologically informed approach linking representational practices to material structures is Michel Foucault’s (1980) thinking about what he called the *dispositif*.3Translated into English as “apparatus”, *dispositif* is described by Agamben (2009:14) as “anything that has in some way the capacity to capture, orient, determine, intercept, model, control, or secure the gestures, behaviors, opinions, or discourses of living beings.” Better known in German-language scholarship (e.g. Bührmann and Schneider 2008), dispositif (or dispositive) analysis, this is something which Anglophone scholars have almost completely overlooked in favour of his notion of *discourse*. (A rare but recent exception is Jannis Androutsopoulos’ (2022) study of mask-requirement signs during the COVID pandemic.). Foucault (1980: 194) explains what he means by *dispositif* in the following way:

What I am trying to pick out with this term is, first of all, a thoroughly heterogeneous ensemble consisting of discourses, institutions, architectural forms, regulatory decisions, law, administrative measures, scientific statements, philosophical, moral and philanthropic proportions – in short: the said as much as the unsaid.

Notably, Foucault hereby calls attention to some quite tangible materialities – buildings, for example – as well as infrastructural or systemic materialities like science, the law, and administration. Once again, textual or representational practices are only ever part of the bigger picture.

In the scenario depicted in Figure 4, the texts themselves – the protest signs – are evidently part of a much larger dispositif – not least of which are the legal “instruments” (themselves written documents) about which these people are protesting. Besides, there is also so much more at stake: from the political systems of government and legislation to the buildings and other infrastructural arrangements where everything takes place.

### 2.3 Mediated discourse analysis

Coming somewhat closer to language studies, another important precedent for engaging with the relationship between language and materiality is to be found in mediated discourse analysis. This is an approach initiated by Ron Scollon (2001) but also developed extensively by Rodney Jones and Sigrid Norris (2005). The practice of shopping offers itself as a quintessential example of what mediated discourse analysis has in mind; in the grocery store depicted in Figure 5, for example, we have what Scollon (2001: 3–4) refers to as a “site of engagement”:

A mediated action occurs in a social space which I [call] a ‘site of engagement’. This is the real-time window that is opened through an intersection of social practices and mediational means (cultural tools) that make that action the focal point of attention of the relevant participants.

Here, the act of shopping is just the focal point in a constellation or “nexus” of semiotic, embodied, material, and spatial features – or “mediational means”. So, for example, as in the image, the written text hanging above the aisle generates its meaning only in conjunction with its specific emplacement, the architectural arrangement of the space, the shelved products, the labels on these products, the shopping trolley, the embodied actions of shoppers, and so on. Discourse is inherently and unavoidably always mediated by other non-discursive (e.g. material) tools.

### 2.4 Social semiotics

Finally, and closely related to mediated discourse analysis in terms of its intellectual origins and major proponents, social semiotics is another language-rooted approach which also explicitly attends to materialities. Emerging from the systemic functional linguistics of Michael Halliday (see Eggins 2004 for an overview), social semiotics has been reconceived as a fully multimodal approach through the work of Gunther Kress (e.g. 2010) and Theo van Leeuwen (e.g. 2005). In this regard, van Leeuwen (2011: 1) offers the following neat summary of its core objectives:

Social semiotics … has three dimensions: (1) the study of semiotic resources and their histories; (2) the study of semiotic practices, of the use of semiotic resources in their specific social, cultural and historical contexts, together with the discursive practices that evaluate, teach, explain and control these uses; and (3) semiotic change, the exploration and development of new semiotic resources and new semiotic practices.

The notion of “semiotic resources” used here is very similar to Scollon’s “mediational means”: the many different ways meaning is constituted multimodally through, for example, words, images, colours, typography, material objects, and spatial settings. It is in this way that a complex social action – like the anti-racism campaign shown in Figure 6 – comes to be organized, produced and potentially understood.

In this case, the text is patently constituted through the combination of discursive and non-discursive resources such as words, artefacts, shapes, colours, setting, and so on. In this case, the verbal message – *Jetzt Gegen Rassismus* (‘against racism now’) – is accomplished with, and underscored by, the large concrete X object which is raised off the ground by large concrete blocks and placed in the large Bahnhofplatz (‘station square’) of Bern, a major thoroughfare for many of the city’s residents and workers.

As I say, failing to acknowledge these types of well-established precedents is an unfortunate oversight on the part of Shankar and Cavanaugh (2012; Cavanaugh & Shankar 2017) in their effort to establish the relationship between language and materiality. Notwithstanding, *language materiality* is certainly a very productive framework for upholding the inseparability of language and materiality (again, cf. Barad 2003; also Miller 2005). In doing so, *language materiality* also helpfully specifies two different but interrelated kinds of materiality: (a) the way words and things *co-signify* and are mutually dependent in the making of meaning; and (b) the ways words structure (and are structured by) material conditions, economies, and power relations. I like to think of these two materialities as being concerned with, respectively, little “m” materialities and big “M” materialities. In this regard, I am also deliberately following James Gee’s (1990) well-known formulation for distinguishing between linguistic discourse and Foucauldian discourses.

With this two-part analytical distinction in mind, I now offer some brief but illustrative examples drawn from my own research – two examples of each type of materiality. In presenting these examples, I also add two qualifying details for helping to understand *language materiality* even further: first, how little “m” materialities can function as transmodal actions, and second, how big “M” materialities can function as a form of symbolic violence.

## 3 Little “m” materialities as transmodal action

Simply put, *transmodalization* describes the process by which one semiotic mode is rendered or “translated” into another semiotic mode. I use the term transmodalization (cf. Newfield 2014) in ways which are closely akin to Kress’ (1997) notion of “transduction” and Jakobson’s (1959: 233) much earlier “intersemiotic translation”, which he glossed as “an interpretation of verbal signs by means of signs of nonverbal sign systems”. Practices of transmodalization have been analytically very important for my research on elitist discourse: the way language functions in the production and maintenance of class privilege. Throughout this work, I have been struck by the central interplay between words and things as a semiotic (or metasemiotic) tactic for producing and performing status, distinction, and prestige. The first case study is a case in point.

**Figure 7:**
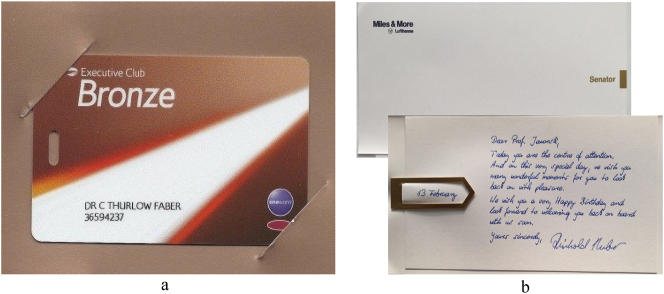
(a and b) Word-things and thing-words (photos mine).

The case study in question was first published in Cavanaugh and Shankar’s (2017) volume on *language materiality*; it focused on what my colleague Adam Jaworski and I called “thing-words” and “word-things”. In this regard, we were interested in two transmodal processes: the way things are often “wordified” and the way words are often “thingified” (Thurlow and Jaworski 2017). The examples we used came from our personal experience of airline frequent-flier programmes. In my case, it was my having been awarded Bronze membership by British Airways along with the plastic, bronze-coloured luggage tag shown in Figure 7a. In Adam’s case, it was the birthday card in Figure 7b he had received from Lufthansa – a special recognition reserved only for Gold members.

In the first instance, stuff – the notion of metal or metal-ness – is made meaningful through its verbal narration or wordification. Specifically, this process entailed the discursive transformation of material trinkets – plastic tags – into something desirable and valuable. At the same time, of course, the words are made to seem consequential and worthy. In the second instance, Adam’s birthday card is a superb example of what Norman Fairclough (1989: 62) calls “synthetic personalization” whereby corporations create the appearance of treating customers as if they were special individuals.

There are any number of semiotic resources – or mediational means – at work in these little texts, but I want to focus on just the one: the use of the handwriting in Adam’s birthday card. This is also a material and materializing performance of special attention: a handwritten message signed by someone official in blue ink. These are thus words which have been thingified, turned into both an object and a commodity (cf. Shankar and Cavanaugh 2012). Once again, it is almost impossible to separate the verbal from the nonverbal, the linguistic from the material. The materiality of the blue ink, along with the accompanying gold-coloured metal clip, is a perfect example of how little “m” materialities generate meaning in their own right. So too, is the high-quality card stock used, which brings me nicely to my next case study while also returning to some high-end dining.

**Figure 8:**
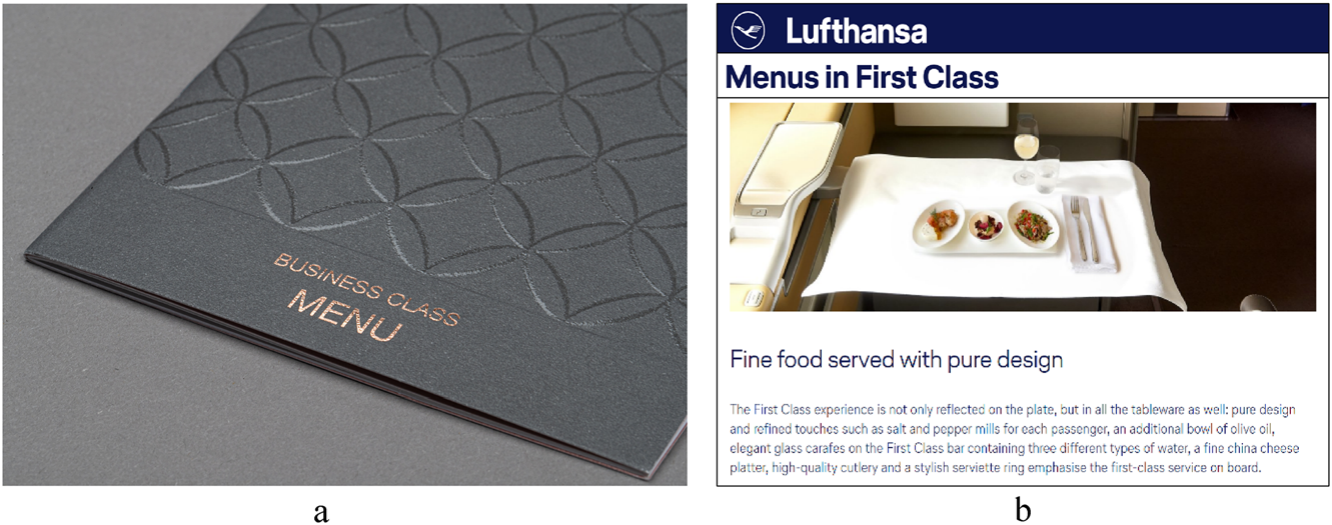
(a and b) Business-class menu and some first-class dining promotion (photos mine).

Language scholars often like to look at the words on a page, but seldom do they consider the page itself - in other words, the paper. With this in mind, and within the same domain of elitist discourse, I have examined material tactics used in the production of business-class menus (see Thurlow 2020). Here, the main objective was to focus on the tactile, textural, and other material properties which help produce feelings of distinction, both literal and figurative. An example is shown in Figure 8a. While menus obviously depend on words (which are themselves wordifications of food), they also have an inherent materiality (e.g. their shape, size, weight, surface textures), all of which functions semiotically in its own particular ways. In this sense, therefore, *language materiality* helps direct attention not only to the multimodality of texts but also to their multisensoriality.

As part of the same research project, and working with a former student assistant, I also examined business-class plateware: the cutlery, dishes, napkins, and tablecloths used for staging super-elite dining experiences (see Thurlow and Haudenschild 2022). In fact, a huge amount of marketing attention is paid to these “premium” meals which is why, of course, the menus are so elaborate. The same excessive attention is also paid to plateware with untold advertising copy generated to describe and promote these material details – this stuff. An example is shown in Figure 8b which shows an extract from a major airline’s website where “fine food” and “pure design” are coupled; as such, the stuff people eat and the stuff they eat with are being mingled. Once again, transmodal actions are at work: the narration of material objects and the materialization of status through the deployment of things.

In sum, these various kinds of little “m” materialities reveal something essential about elite discourse but also about *language materiality* in general. Everywhere we find the strategic, transmodal interplay of semiotic resources. There is also often an obfuscating toggle between linguistic practices and material practices, between words and things, or ideas and stuff. In fact, this transmodal toggling is precisely what makes elitist discourse so persuasive and effective – so powerful.

## 4 Big “M” materialities as symbolic violence

**Figure 9:**
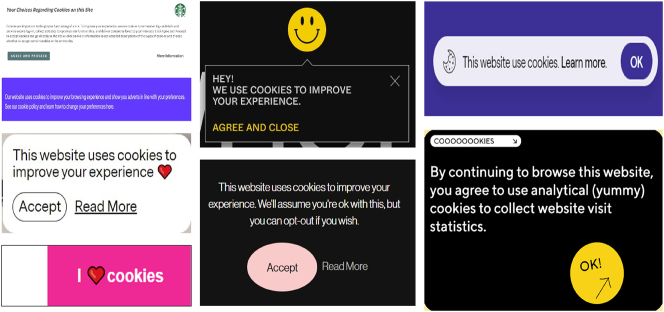
Montage of cookie consent notices (various sources).

In speaking of power, I turn now to the first of two case-study examples of big “M” materialities. These are instances where *language materiality* functions as an exercise not only of symbolic power but more specifically of *symbolic violence*. In this regard, I follow Bourdieu and Wacquant (1992) in using the term symbolic violence to describe how people allow words to charm and control them even when this may be against their own best interests. Put differently, control over people is achieved through seduction rather than by force. To exemplify this, I offer some evidence which appeared in a recent piece about language work in the movies (Thurlow 2022b) where, amongst other things, I considered the power of little texts like subtitles and closed-captions. First, however, and to demonstrate why little texts are often big in their material consequences, I used the example of cookie consent notices, some examples of which appear in the Figure 9 montage.

Lara Portmann (2022) has written more extensively about the way cookie consent notices function both pragmatically and ideologically; her thinking is also heavily informed by Rodney Jones’ (2020) perspective on consent notices in the context of digital surveillance culture. In my own paper, meanwhile, the point I wanted to make was that cookie consent notices exemplify the how big “M” materialities work in/through even the most banal, seemingly harmless little texts. These specific little texts surreptitiously manage our digital interactions and thus shape our everyday lives, even if we barely notice them or hurriedly dismiss them.

Under European Union law, website owners are obliged to secure consent before recording any user information. Cookie consent notices have therefore proliferated. Needless to say, however, few of these notices draw attention to the precise nature of the information being recorded nor to the legal implications in consenting. Most often, the verbal content is strategically designed to seem playful and innocuous – precisely to discourage users from looking more closely at the small print and possibly adjusting their preferences. This helps explain the heart and smiley-face emojis, the light-hearted, informal language, and the metaphoric use of edible cookies; it is all about persuading users that the consenting act is quite ordinary, uncomplicated, and innocuous. Note also the tactical use of colour and other salient design features to encourage acceptance: the bright yellow “OK” buttons or pink “accept” button.

Ultimately, what we see here is how material conditions such as the legal system shape the language and communication used. But we also see how this language is used to manage people and to enforce the law. Users regularly allow themselves to be charmed and persuaded this way which is how symbolic power *and* symbolic violence work.

**Figure 10:**
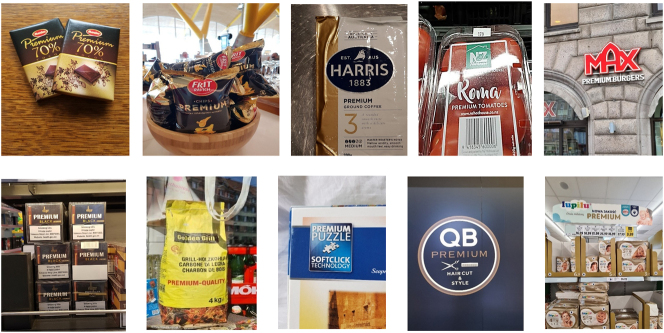
Montage of “premium” products (photos mine).

Sometimes, big “M” materialities depend on quite elaborate texts and communicative practices; even little texts can be relatively sophisticated in their design. At other times, though, the messages can be a lot more succinct and efficient, perhaps even just a single word. One of my favourite cases in point is the word “premium”, which was the focus of another of my studies (see Thurlow 2021). This seemingly “empty” word or catchphrase crops up all over the place, meaning everything and nothing at the same time. In Figure 10, I offer just a handful of examples from the many I have been collecting over the years: premium chocolates in Sweden, premium chips in Spain, premium coffee in Australia, premium tomatoes in New Zealand and burgers in Denmark, premium cigarettes and premium BBQ charcoal in Switzerland, premium puzzles in Germany and premium haircuts in Hong Kong, and then premium diapers (or nappies) in Poland.

As I understand see it working in the world, the word or label “premium” is an effort to persuade people to seek something a little extra or a little more – especially a little extra or a little more compared to other people. And, as marketers know well, people will pay good money to feel like they are better off. As such, these seemingly harmless language games are another example of symbolic violence: words used to manipulate people even when it is against their best interests. This may be a matter of their being tricked into paying more or being persuaded into relentless social comparison; either way, we have here a one-word message which nicely exemplifies how big “M” materialities can be realised in or through language.

## 5 Summing up: trash talk, storied matter, and the stuff of words

Given the examples I have offered, it might well appear that the stuff of words is a matter only of written language; this is most certainly not the case. Words are not only concrete but also bodily; when people write, it is ink that is scratched, struck or pressed onto paper, just as when they speak it is sound waves that beat against the eardrums and resonate through the body. Partly with this in mind, but also turning more concretely to the classroom, I want to start summing up by way of a small “trash talk” exercise. In doing so, I shift attention from excessive consumption practices to practices of wasting – a new direction in my own research agenda but also a new domain for language scholars (see Thurlow 2022c).

This particular exercise arises from a workshop I have been running for some years but is a trash-related iteration developed together with my doctoral students, Charmaine Kong, Alessandro Pellanda and Laura Wohlgemuth.4In June 2024, we used this exercise in workshops at the 15th Linguistic Landscape conference (Wellington, New Zealand) and the 25th Sociolinguistics Symposium (Perth, Australia). Several participants commented on the instructional potential of the exercise in language-learning classes. The objective of the exercise is to encourage participants to surface materialities – both small and big – by generating a short object biography (Kopytoff 1986), thinking through the way discarded matter might engage them if they imagine the object speaking to them. In doing so, participants are also invited to reflect on the opportunities and limits of relying on language for understanding things and, specifically, waste. The exercise is thus one which combines the “micropolitics” (Hawkins 2006) of noticing waste while also highlighting how *storied matter matters* (Oppermann 2013).5“Storied matter matters” has become something of a mantra or organizing principle for my research team and me in seeking to uphold both the central role of representation in waste/wasting and its inherently non-presentational nature too.


Exercise: *Object interviewing*

Participants are invited to bring with them (in a plastic bag or jar) an item of trash from their home or, preferably, the instructor supplies a selection of items retrieved from nearby trash cans (e.g. in the building or on campus). Allowing time for individual reflection and perhaps writing, participants are given the following instructions or prompts:

1. Start by taking a photo of the object.
2. Now answer the following questions: What is it? How might you describe it? How does it feel, smell, etc.?
3. What else can you already know about it? What is it made of? Who is it for? Who uses it?
4. Can you imagine how it was produced – where and by whom? What did it cost to make and/or to buy?
5. What else might you want to know about its biography?
6. Now imagine the object speaking for itself. What might it say? What might it want you to know?
7. Finally, take another picture of your object. Does this second photo look like the first? What changed? Did the interview change your relationship with the item?

**Figure 11:**
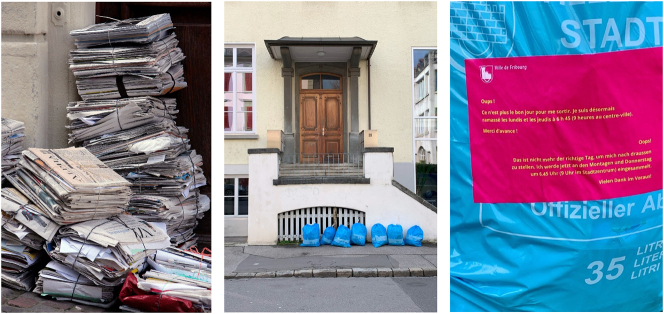
(a–c) Snapshots of garbage day in Switzerland (photos Alessandro Pellanda).

Waste is a quintessential case of *language materiality* at work in the world, another obvious example of which is illustrated in Figure 11a. In this snapshot of Swiss life, we see newspapers and advertising brochures neatly bundled and placed for curbside pick-up and recycling. This is language – in its printed, written form – quite tangibly materialized. Words are thus thingified and, like so many things, must be disposed of.

Waste presents itself as a fascinating but challenging conundrum, however. It is most readily known to us as things and stuff – as little “m” materialities. But waste is also shot through with language: it is, for example, with words that we name and categorize the things which have value, or which have no value – the things we therefore keep and the things we throw away. Words not only help define what waste is and is not, but also help produce, maintain, and regulate the everyday practices of waste-making – both small scale and large scale.

In Figure 11b, I offer another snapshot from the streets of Switzerland. This photo was taken by Alessandro Pellanda (see Thurlow et al. 2022). The scene shows garbage collection day in Fribourg: a neat row of rubbish in the official bright blue garbage bags. This is not just a scene of waste management, however. We regard these banal moments of wasting as evidence for the way waste sometimes functions as a semiotic resource in its own right. These bags are little “m” materialities for people to perform their identity as responsible citizens; collectively, two times a week these material practices help communicate and sustain a key mythology of Swissness.

Notwithstanding the obvious materiality of waste, language always plays a central role in waste making. Much of this discursive mediation takes place behind the scenes, but sometimes it makes itself known – as in Figure 11c where we find a friendly reminder-cum-warning pasted onto a garbage bag left outside at the wrong time of day. Translated from French and German, the message reads: *Oops! This is not the right day to take me out. I’m only collected on Mondays and Thursdays at 6:45 (09:00 in the city center). Thanks in advance.* In yet another little text, this is a glimpse into the far more extensive documentary regimes (Cavanaugh 2022) which underwrite the management and regulation of domestic waste in the city. In other words, here we have evidence of the *dispositif* – the political, institutional and financial-juridical systems which structure waste practices. This is big “M” materialities at work around some otherwise little “m” materialities. Nor is the synthetically personalized style of the little text unimportant. In fact, this brings me full circle in many ways. Like the wheelchair text I started with, the symbolic power at work here also entails a kind of symbolic violence. Here we find words acting on people – controlling them – in seemingly benign, friendly ways, but with very real, concrete, *material* consequences.
